# The Impact of Melatonin and NLRP3 Inflammasome on the Expression of microRNAs in Aged Muscle

**DOI:** 10.3390/antiox10040524

**Published:** 2021-03-27

**Authors:** Ramy KA Sayed, Marisol Fernández-Ortiz, José Fernández-Martínez, Paula Aranda Martínez, Ana Guerra-Librero, César Rodríguez-Santana, Tomás de Haro, Germaine Escames, Darío Acuña-Castroviejo, Iryna Rusanova

**Affiliations:** 1 Department of Anatomy and Embryology, Faculty of Veterinary Medicine, Sohag University, Sohag 82524, Egypt; ramy.kamal@vet.sohag.edu.eg; 2 Centro de Investigación Biomédica, Departamento de Fisiología, Facultad de Medicina, Instituto de Biotecnología, Parque Tecnológico de Ciencias de la Salud, Universidad de Granada, 18016 Granada, Spain; sol92@correo.ugr.es (M.F.-O.); josefermar@ugr.es (J.F.-M.); ampaula@correo.ugr.es (P.A.M.); aguerit@ugr.es (A.G.-L.); cesar@correo.ugr.es (C.R.-S.); gescames@ugr.es (G.E.); dacuna@ugr.es (D.A.-C.); 3 UGC de Laboratorios Clínicos, Hospital Universitario San Cecilio, 18016 Granada, Spain; tomas.haro.sspa@juntadeandalucia.es; 4 CIBERfes, Ibs. Granada, 18016 Granada, Spain; 5 Department of Biochemistry and Molecular Biology I, Faculty of Science, University of Granada, 18071 Granada, Spain

**Keywords:** microRNAs, melatonin, NLRP3 inflammasome, NF-kB, aging

## Abstract

Muscular aging is a complex process and underlying physiological mechanisms are not fully clear. In recent years, the participation of the NF-kB pathway and the NLRP3 inflammasome in the chronic inflammation process that accompanies the skeletal muscle’s aging has been confirmed. microRNAs (miRs) form part of a gene regulatory machinery, and they control numerous biological processes including inflammatory pathways. In this work, we studied the expression of four miRs; three of them are considered as inflammatory-related miRs (miR-21, miR-146a, and miR-223), and miR-483, which is related to the regulation of melatonin synthesis, among other targets. To investigate the changes of miRs expression in muscle along aging, the impact of inflammation, and the role of melatonin in aged skeletal muscle, we used the gastrocnemius muscle of wild type (WT) and NLRP3-knockout (NLRP3^−^) mice of 3, 12, and 24 months-old, with and without melatonin supplementation. The expression of miRs and pro-caspase-1, caspase-3, pro-IL-1β, bax, bcl-2, and p53, was investigated by qRT-PCR analysis. Histological examination of the gastrocnemius muscle was also done. The results showed that age increased the expression of miR-21 (*p* < 0.01), miR-146a, and miR-223 (*p* < 0.05, for both miRs) in WT mice, whereas the 24-months-old mutant mice revealed decline of miR-21 and miR-223 (*p* < 0.05), compared to WT age. The lack of NLRP3 inflammasome also improved the skeletal muscle fibers arrangement and reduced the collagen deposits compared with WT muscle during aging. For the first time, we showed that melatonin significantly reduced the expression of miR-21, miR-146a, and miR-223 (*p* < 0.05 for all ones, and *p* < 0.01 for miR-21 at 24 months old) in aged WT mice, increased miR-223 in NLRP3^−^ mice (*p* < 0.05), and induced miR-483 expression in both mice strains, this increase being significant at 24 months of age.

## 1. Introduction

The aging process is accompanied by an increase in chronic inflammation and oxidative stress that weaken various organs including skeletal muscle, and yield a condition of frailty. The mechanism(s) involved in the regulation of the inflammatory process in skeletal muscle are not entirely clear, but recent data point to the participation of NF-kB and NLRP3 (the nucleotide-binding and oligomerization domain, leucine-rich repeat, and pyrin domain-containing 3) inflammasome in skeletal muscle aging [1,2,3]. NF-kB and NLRP3 inflammasome are the main components of the innate immune system, the first line of the host defense in response to harmful stimuli, such as internal changes related to age or environmental irritants.

NLRP3 inflammasome contains a triple protein complex: the sensor NLRP3, the adaptor ASC (apoptosis-associated speck-like protein containing a CARD), and the effector pro-caspase-1 [4]. A two-signal model for NF-kB/NLRP3 inflammasome activation, which included increased oxidative stress and mitochondrial dysfunction, which happen for example during aging, has been proposed [4,5,6]. Together with the decreased capacity to synthesize new proteins and less cell differentiation, these mechanisms lead to muscle dysfunction and sarcopenia. microRNAs (miRs) rise as important epigenetic regulators of a wide range of physiological and pathological processes, including aging and age-related diseases, and they play a critical role in the regulation of the skeletal muscle proliferation, differentiation, and apoptosis [7,8,9]. The expression of miRs changes throughout life, and it can be modified by external factors such as nutritional status [10] and physical exercise [11], suggesting that the formers could be perfect targets for regulating muscular state.

miRs are small non-coding molecules containing approximately 18-22 nucleotides, and are able to regulate the expression of their target mRNAs at the post-transcriptional level [12,13]. Each individual miR is capable of targeting diverse mRNAs and, at the same time, various miRs can act on each individual mRNA [14], forming a complex gene regulatory network and controlling numerous biological processes throughout life, including priming and activation of inflammasomes [5]. Currently, the miR’s studies are gaining high relevance for both diagnosis and searching for specific treatments in a wide range of diseases [15,16] However, the knowledge of miRs expression changes and their relationship with changes leading to muscular age is yet scarce. Several previous works have demonstrated that miR-21, miR-146a, and miR-223 are differentially expressed with age [17] and they are related to the control of innate immunity and inflammation [18,19,20,21]. The particular interest in the miR-483 study is based on its participation in the regulation of melatonin synthesis [22].

Melatonin (N-acetyl-5-methoxytryptamine, aMT) is a hormone involved in multiple functions, including antioxidative, anti-inflammatory, and immunomodulatory, and mitochondrial protective effects [23,24,25,26]. It is originally isolated from the pineal gland and is considered as a regulator of circadian rhythms and seasonal breeding [27]; however, it is currently known that melatonin is widely produced and distributed in all tissues and organs, including the skeletal muscle [28]. Recent studies determined that oral melatonin supplementation preserves normal structure and muscular activity in aged mice. These studies also confirmed the NLRP3 inflammasome implication in muscular aging [26,29]. Although there are some miRs involved in the regulation of the inflamma-pathways and of the NLRP3 inflammasome activity, it is not known whether their expression changes in aged muscle and what effect melatonin can exert on their expression with age.

Due to the connection between miRs, innate immunity, and melatonin in age, we considered it worthwhile to analyze the expression of miR-21, miR-146a, miR-223, and miR-483 in the gastrocnemius muscle of young (3-months old), early aged (12-months old), and old-aged (24-months old) wild type, and NLRP3-knockout mice, as well as the effect of melatonin supplementation. The results of this study will clarify new aspects of the pathophysiology of skeletal muscle during aging.

## 2. Materials and Methods

### 2.1. Experimental Animals

Female NLRP3-knockout mice NLRP3^−/−^ (B6.129S6-Nlrp3tm1Bhk/J) on the wild-type C57BL/6J background (> 10 backcrosses) were purchased from The Jackson Laboratory, (Bar Harbor, ME, USA). The NLRP3^−/−^ mice were bred to wild-type C57Bl/6J mice according to instruction of The Jackson Laboratory. Mice were housed in the pathogen-free barrier Granada University’s animal facility under controlled conditions of humidity and 12:12 h light/dark cycle, at 22 °C ± 1 °C, and with chow and tap water ad libitum. All experiments were carried out in accordance with the guidelines of the University of Granada’s Ethical Committee (CEEA 462-2013); the Ethical Committee of the Junta de Andalucía, Spain (no. 05/07/2016/130), based on the directive 2010/63/EU of the Spanish Protection Guide for Animal Experimentation (R.D. 53/2013); and the European Convention for the Protection of Vertebrate Animals used for Experimental and Other Scientific Purposes (CETS # 123).

WT and NLRP3^−^ animals were divided into five groups (n = 5 animals per group): (1) young (Y, 3-months old); (2) early aged (EA, 12-months old); (3) early aged treated with melatonin (EA + aMT); (4) old-aged (OA, 24-months old); (5) old-aged treated with melatonin (OA + aMT). Melatonin (aMT) was orally administered through chow’s pellets to ensure that each mouse receives 10 mg/kg body weight/day for two months before sacrifice. This dose of melatonin was reported previously as dose with significant anti-aging benefits in mice [30]. Adding melatonin to the chow’s pellets was performed in the Diet Production Unit facility of the University of Granada and its amount was checked by HPLC (data not shown).

### 2.2. Total RNA and miRNA Extraction and qRT-PCR

For total RNA extraction, including miRs, tissue miRNeasy Mini kit was used (ref. 217004, Qiagen, Madrid, Spain) following the manufacturer’s instructions. Frozen (−80 °C) muscle tissue (50–100 mg) was homogenized and lysate in 700 μL of QIAzol Lysis Reagent. After 5 min incubation at room temperature, 140 μL of chloroform were added, and the mixture was incubated for 3 min. The lysate was separated into aqueous and organic phases by centrifugation for 15 min at 12,000× *g* at 4 °C. Next, 1 volume of 70% ethanol was added to the aqueous phase and the solution was passed through the RNeasy Mini spin column, using RNeasy Min Elute Cleanup Kit (ref. 74204, Qiagen), in order to make the small RNAs, including miRs in the flow-through. RNeasy Mini spin columns were conserved for further extraction of total RNA. The fraction of small RNAs was washed with 100% ethanol and passed thorough RNeasy MinElute spin columns. Finally, small RNAs were eluted with 14 μL of RNase-free water. For extraction of total RNA, RNeasy Mini spin columns were washed with buffer provided by the manufacturer, and finally, total RNA was eluted with 50 μL of RNease-free water. The quantity and quality of RNA were determined in a NanoDrop by 260:280-nm ratio absorbance and gel electrophoresis, respectively.

Total of 200 ng of total RNA was used for cDNA synthesis with the qScriptTM cDNA SuperMix kit (Quanta Biosciences, Gaithersburg, MD, USA). qRT-PCR was performed with the iTaq SYBR Green Supermix kit (Bio-Rad Life Sciences, Madrid, Spain). The PCR program was initiated with 10 min at 95 °C followed by 40 thermal cycles, each consisting of 15 s at 95 °C and 30 sec at 55 °C. Beta-actin housekeeping was used as an endogenous reference gene; a no template-free (water) reaction was used as negative control to determine any contamination, and untreated NLRP3^+^ was used as a calibrator sample. Output data were analyzed with the MxPro QPCR software (Agilent Technologies, Barcelona, Spain) according to the standard curves elaborated with different amounts of cDNA. All PCR were performed in a Stratagene Mx3005P QPCR System (Agilent Technologies, Barcelona, Spain). Forward and reverse primers used for detection of transcripts are listed in Supplementary Table S1.

For determination of miRs expression two steps analysis was performed. Reverse transcription was carried out in 10 ng of small RNA, including miRs, using TaqMan^®^ microRNA Reverse Transcription Kit (Life Technologies, Thermo Fisher Scientific, Madrid, Spain) and Taq Man microRNA assays specific for each miR (miR-21-5p, assay ID 000397; miR-146a-5p, assay ID 000468; miR-223-3p, assay ID 002295, and miR-483-5p, assay ID 002338). Small nuclear (snRNA) U6 was used as endogenous control (assay ID 001973). The PCR was carried out as the following conditions: 16 °C for 30 min, 42 °C for 30 min, 85 °C for 5 min, and then kept to 4 °C. Quantitative real-time polymerase chain reaction (qRT-PCR) was performed in a final volume of 20 μL, containing 10 μL of TaqMan^®^ Universal Master Mix 2x with uracil-N-glycosylase (UNG) (Applied Biosystems), 1 μL of 20x specific TaqMan Small RNA Assay (Life Technologies), 7.67 μL RNase-free water, and 1.33 μL of the RT product. Real-time reactions were carried out at 95 °C for 10 min, followed by 40 cycles at 95 °C for 15 s and at 60 °C for 60 s. All reactions were run in triplicate in a Stratagene Mx3005P QPCR System (Agilent Technologies, Barcelona, Spain) with automatic baseline setting. For data analysis of PCR, experiments with fixed comparative threshold (Cts) less than 33 will be selected. Data were analyzed using the SDS 2.3 and RQ Manager 1.2 software and the relative expression levels of each miRNA were calculated using 2^−ΔΔCT^ method (ΔCt target gene −ΔCt control gene).

### 2.3. Histological and Morphometric Analysis of the Collagenous Tissue

WT and NLRP3^−^ mice were weighted and anaesthetized via intraperitoneal injection of equithesin (1 mL/kg). The animals were transcardially perfused with a warm saline and a solution of freshly prepared trump’s fixative (3.7% formaldehyde plus 1% glutaraldehyde in saline buffer). The gastrocnemius muscle was removed, and excessive connective tissues were dissected. The muscle was fixed in the trump’s fixative, divided into two halves at the mid-belly and then was immersed in Bouin’s solution for histological examination.

After proper fixation, cross samples of the gastrocnemius muscle were extensively washed in ethanol 70%, dehydrated in ethanol ascending graded concentrations, cleared in xylene, and embedded in paraffin wax. Sections of 4-μm thick were cut with an SLEE Mainz Cut 5062 microtome, dewaxed in xylene, rehydrated in ethanol descending series of ethanol (100%, 95%, 80%, and 70%), and washed with distilled water. The sections were then stained with hematoxylin and eosin stain [31] for general histological examination, Crossmon’s trichrome stain [32] and Van Gieson stain [33] for differentiation of collagenous connective tissue and muscle fibers. All of these stains were applied in accordance with Bancroft’s theory and practice of histological techniques [34]. Following staining, the sections were dehydrated again in an ascending series of ethanol (70%, 95%, and 100%), cleared in xylene, and mounted with DPX. The sections were examined using a Carl Zeiss Primo Star Optic microscope, and digital images were acquired by a Magnifier AxioCamICc3 digital camera (BioSciences, Jena, Germany).

Morphometric analysis of the collagenous tissues was performed on the images of Van Gieson-stained sections at a ×40 magnification, using the Image J processing software. The percentage of the area occupied by the collagen to the total muscular field of the microscope was estimated. Collagen analysis was applied on 15 randomly selected sections of the gastrocnemius muscle per animal, and was conducted by two double-blinded operators, comparing the obtained data subsequently. The percentage of the collagen was presented as mean ± SEM.

### 2.4. Statistical Analysis

All statistical analyses were carried out using GraphPad Prism 6.0 software (GraphPad Software, San Diego, CA, USA). One-way ANOVA with a Tukey´s post-hoc test was used to compare the differences between experimental groups. Statistical analyses between groups were performed using an unpaired two-tailed t test. For analysis of correlations between parameters Spearman test was used. All data of independent experiments were expressed as mean ± standard error of the mean (S.E.M.). *p* < 0.05 was considered as statistically significant.

## 3. Results

### 3.1. Age-Dependent miRs Expression Changes in the Gastrocnemius Muscle of Wild Type and NLRP3^−^ Mice

**Figure 1:**
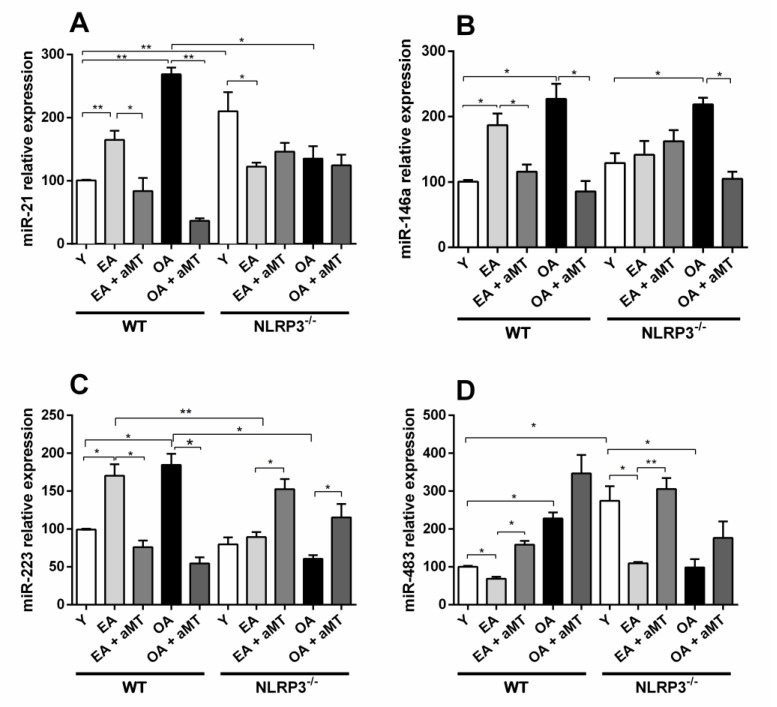
Age-related microRNAs expression changes in the gastrocnemius muscle of wild type and NLRP3^−^ mice and melatonin treatment. Relative miR-21, miR-146A, miR-223, miR-483 expression from gastrocnemius muscle of young (Y), early aged (EA), early aged with melatonin (EA + aMT), old-aged (OA), and old-aged with melatonin (OA + aMT) of wild type (WT) and NLRP3− mice (**A**–**D**). miR-21, miR-146a and miR-223 expressions increased in the gastrocnemius muscle of mice during aging; melatonin administration managed to decrease ones. The suppression of NLRP3 inflammasome activation was related with decreased miR-21 and miR-223 levels, without changes in miR-146a. In NLRP3^−^ mice, melatonin administration did not influence miR-21 expression, reduced miR-146a levels in OA animals and increased miR-223 expression (**A**,**B**,**C**). miR-483 expression was declined in early aged WT mice and increased in the old-aged animals. Young NLRP3^−^ mice had higher miR-483 levels compared to WT ones, and their expression decreased with age. miR-483 expression levels were increased by melatonin in all groups of WT and NLRP3^−^ mice (**D**). Data were calculated using the 2^−ΔΔCt^ method. The expression levels were normalized against U6 snRNA. Data are presented as means ± standard error of the mean (*SEM*). Comparisons between groups are indicated in the graphs. * *p* < 0.05 and ** *p* < 0.01.

miR-21, miR-146a, and miR-223 were significantly increased in gastrocnemius muscle of WT mice along aging (Figure 1). Concerning miR-483, its expression revealed a decline in early aged WT mice; however, the expression was increased in the old-aged animals (Figure 1). In young NLRP3^−^ mice, the expressions of miR-21 and miR-483 were significantly higher than in young WT ones, and their expression decreased with age, being significantly lower for miR-21 and miR-483 in early aged group and for miR-483 in old-aged group. miR-146a increased in old-aged vs. young NLRP3^−^ animals, and this increase resembles that observed in WT mice during aging. miR-223 expression was not changed throughout life in NLRP3^−^ animals, but the lack of NLRP3 made that expression of miR-223 significantly lower in early aged and old-aged mutant animals compared to the WT mice.

Melatonin administration decreased the expression of the three inflamma-miRs (miR-21, miR-146a and miR-223) in early aged and old-aged WT groups, reducing the levels below those observed in young animals in the case of miR-21 and miR-223 (Figure 1A–C). An opposite effect of melatonin administration was observed on miR-223 expression in EA + aMT and OA + aMT NLRP3^−^ mice (Figure 1C). In NLRP3^−^ mice, melatonin administration did not influence miR-21 expression but reduced miR-146a levels in OA animals. miR-483 expression levels were increased by melatonin in all groups of WT and NLRP3^−^ mice (Figure 1D).

### 3.2. Age-Related Changes in the Gastrocnemius Muscle Inflammation of Wild Type and NLRP3^−^ Mice

**Figure 2:**
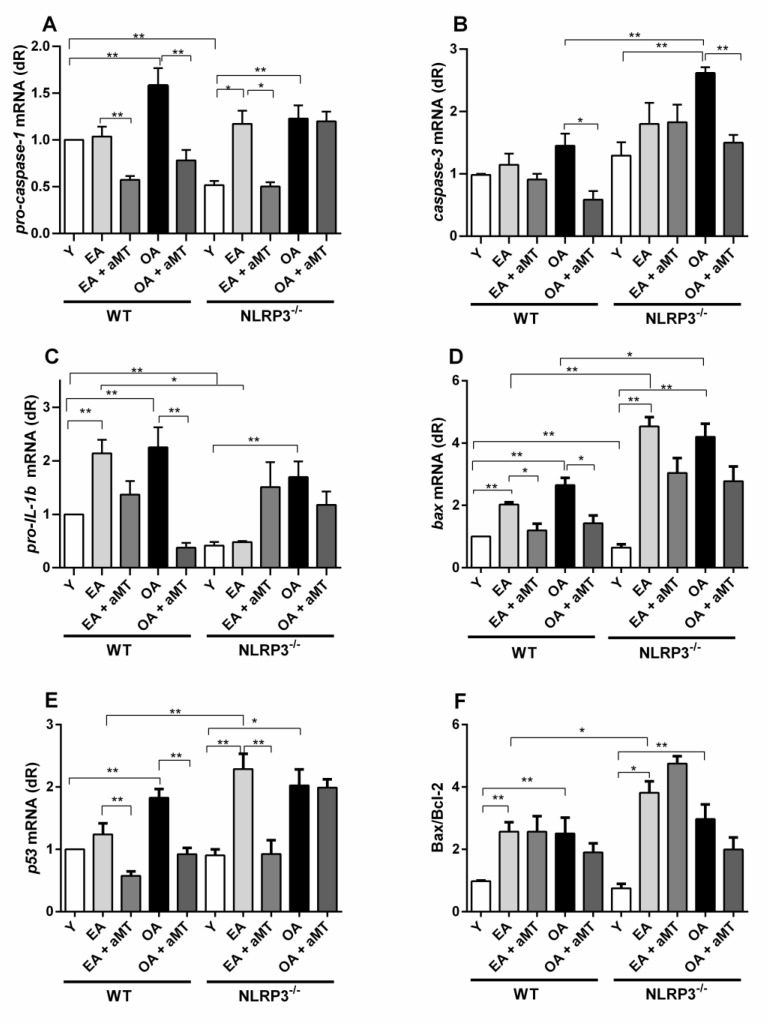
Changes in mRNA expression of the molecular inflammatory and apoptosis parameters in WT and NLRP3^−^ during aging and melatonin supplementation. Experiments were performed in gastrocnemius muscle of young (Y), early aged (EA), early aged with melatonin (EA + aMT), old-aged (OA), and old-aged with melatonin (OA + aMT) of wild type (WT) and NLRP3^−^ mice. Following mRNA expression levels were measured by qRT-PCR analysis: **A**-pro-caspase-1, **B**-caspase-3, **C**-pro-IL-1β, **D**-Bax, **E**-p53, and **F**-ratio Bax/Bcl-2. Increased pro-caspase-1, pro-IL-1β, bax, p53 levels and Bax/Bcl-2 ratio with age in WT animals, were contrarested by melatonin administration in all parameters, except Bax/Bcl-2 (**A**,**C**–**E**). Aging afected the expression of all molecular parameters in NLRP3^-^ animals (**A**–**F**), although the levels of pro-caspase-1, pro-IL-1β and bax were lower in early aged mutant animals compared to the WT ones of the same age (**A**,**C**,**D**). Melatonin administration decreased pro-caspase-1 and p53 expression in EA NLRP3- group (**A**,**E**), as well as reduced caspase-3 levels in OA mutant mice (**B**). Data are expressed as means ± *SEM* (n = 5 animals/group). Comparisons between groups are indicated in the graphs. * *p* < 0.05 and ** *p* < 0.01.

In recent years, evidence has concluded that aging leads to chronic inflammation [35,36]. As expected, inflammatory and pro-apoptotic parameters worsened in mice with age: we found a significant increase in the mRNA expression of pro-IL-1β mRNA, Bax mRNA and Bax/Bcl-2 ratio in early aged WT animals. Pro-caspase-1, pro-IL-1β, Bax, p53, and Bax/Bcl-2 ratio, were significantly increased in old-aged WT animals compared to the young mice (Figure 2).

NLRP3^−^ animals have fewer levels of pro-caspase-1 and pro-IL-1β mRNA expression, as well as the lowest Bax mRNA levels, at 3-months of age, compared with Y WT mice. EA mutant mice presented significant highest expression of pro-caspase-1, Bax, p53 and Bax/Bcl-2 ratio compared to Y mutant animals. Aging affected expression of almost all molecular parameters: old-aged vs. young mice presented significant higher levels of pro-caspase-1, caspase-3, pro-IL-1β, Bax, p53, and Bax/Bcl-2 ratio (Figure 2A,F). These data confirm that age affects the inflammatory markers in NLRP3 mutant animals almost in the same way as of WT mice, although the production of pro-IL-1β was lower in early aged mutant animals compared to the WT ones of the same age (Figure 2C). The lack of NLRP3 had a pro-apoptotic effect by increasing Bax and p53 mRNA expression and Bax/Bcl-2 ratio in EA NLRP3^−^ vs. EA WT mice (Figure 2D,F). With advanced age (OA), a high expression of caspase-3 and increased levels of Bax were found in NLRP3^−^ mice vs. WT OA animals (Figure 2B,D).

Melatonin supplementation significantly decreased pro-caspase-1 mRNA in early aged and old-aged WT, as well as early aged NLRP3^−^ group (Figure 2A). Moreover, caspase-3 and pro-IL-1β mRNA levels were reduced in OA + aMT WT groups (Figure 2B,C). Apoptotic Bax and p53 mRNA expression were decreased in EA + aMT and OA + aMT WT animals, and in EA + aMT NLRP3^−^ mice, but the Bax/Bcl-2 ratio was not influenced by melatonin administration (Figure 2D–F).

### 3.3. Correlation Analysis between miRs and Inflammatory Markers in the Gastrocnemius Muscle of Wild Type and NLRP3^−^ Mice

**Figure 3:**
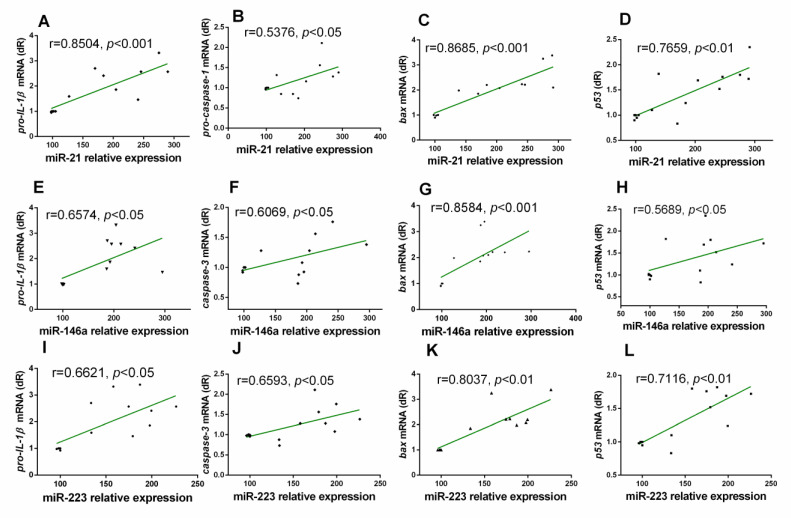
Correlations between miR-21, miR-146a, and miR-223 expression and pro-inflammatory and proapoptotic markers in gastrocnemius muscle of WT mice with aging, calculated using analysis of Spearman correlation coefficient (r). Positive correlations were found between miR-21 expression and pro-IL-1β, pro-caspase-1, bax and p53 levels (**A**–**D**). Possitive correlations were detected between miR-146a expression and pro-IL-1β, caspase-3, bax and p53 (**E**–**H**). Positive correlations were observed between miR-223 and pro-IL-1β, caspase-3, bax and p53 (**I**–**L**).

Looking for correlations between the expression levels of miRs and the molecular inflammatory parameters, we found that, in WT animals, miR-21 had a significant positive correlation with almost all of them: with pro-IL-1β, p53, Bax, and pro-caspase-1 (Figure 3A,D). miR-146a expression had a positive correlation with p53, Bax, pro-IL-1β, and caspase-3 (Figure 3E–H). Additionally, positive correlations were also found between miR-223 and p53, Bax, pro-IL-1β), and caspase-3 (Figure 3I,L).

**Figure 4:**
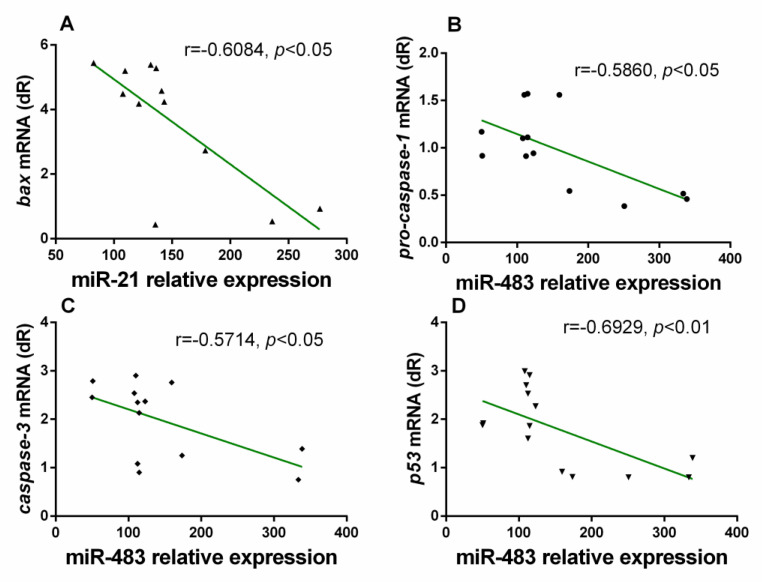
Correlations between relative miR-146a expression and caspase-3 mRNA levels and between relative miR-483 expression and pro-caspase-1 levels in the gastrocnemius muscle of NLRP3^−^ mice with aging, calculated using analysis of Spearman correlation coefficient (r). Negative correlations were found between miR-21 and bax (**A**), as well as negative correlations were detected between miR-483 and pro-caspase-1, caspase-3 and p53 (**B**–**D**).

In NLRP3^−^ mice, we found a negative correlation between miR-21 and Bax. In these mice, negative correlations between miR-483 expression and pro-caspase-1, caspase-3, and p53, were observed (Figure 4). All correlations were found in no-treated mice.

### 3.4. Age-Dependent Changes on the Muscular Structure and Collagenous Content of the Gastrocnemius Muscle in WT and NLRP3^−^ Mice

**Figure 5:**
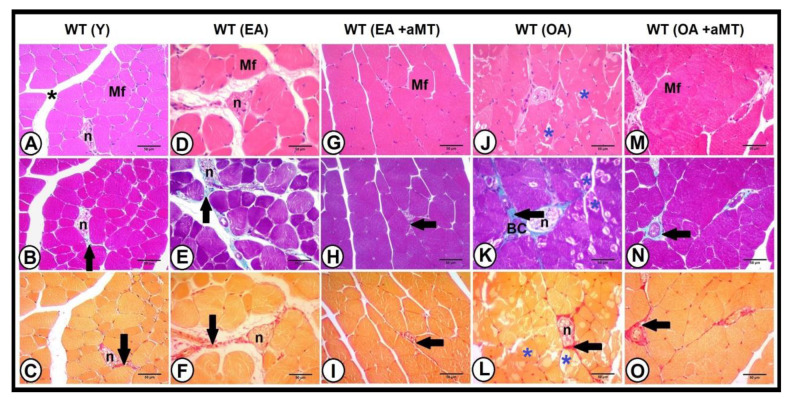
Age-associated changes in the gastrocnemius muscle architecture and the protective effect of melatonin. Photomicrographs of cross section of the gastrocnemius muscles of the young (Y), early aged (EA), early aged with melatonin (EA + aMT), old-aged (OA), and old-aged with melatonin (OA + aMT) WT mice stained with H&E (**A**,**D**,**G**,**J**,**M**); Crossmon’s trichrome (**B**,**E**,**H**,**K**,**N**), and Van Gieson (**C**,**F**,**I**,**L**,**O**) stains. (**A**–**C**) Photomicrographs of the Y mice showing arrangement of muscle fibers in bundles, which are separated by interstitial space (black asterisk) that contains collagenous tissue (arrows) and nerve fibers (n). (**D**–**F**) Photomicrographs of the EA animals revealing widening of interstitial spaces with increased collagenous tissues deposition (arrows). (**G**–**I**) Photomicrographs of the EA animals after melatonin supplementation exhibiting the protective effect of melatonin on reducing muscular interstitial spaces and minimizing collagen deposition (arrows). (**J**–**L**) Photomicrographs of the OA mice illustrating an excessive accumulation of the collagen fibers (arrows) in interstitial spaces around nerves (n) and blood capillaries (BC). Note, presence of necrotic areas (blue asterisk) within muscle fibers of the OA animals. (**M**–**O**) Photomicrographs of OA mice with melatonin treatment depicting the beneficial effect of melatonin on conserving the architecture of muscle fibers (Mf) and reducing collagenous tissue deposition (arrows). Bar = 50 μm.

**Figure 6:**
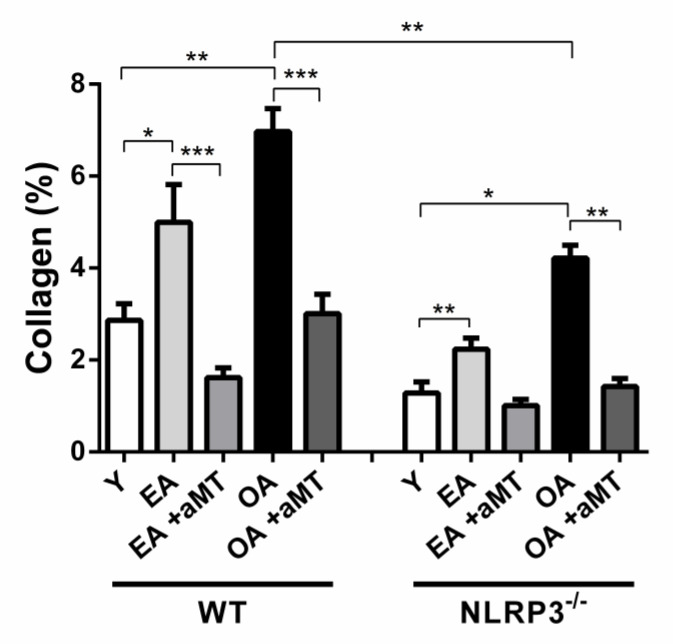
Impact of NLRP3 inflammasome and melatonin supplementation on the collagenous content of the gastrocnemius muscle during aging. Morphometric analysis of the percentage of the collagen in the gastrocnemius muscle of the young (Y), early aged (EA), early aged with melatonin (EA +aMT), old-aged (OA), and old-aged with melatonin (OA+ aMT) WT and NLRP3^−^ mice revealing that aging induced a significant increase in the percentage of the collagen, and this increase was less considerable in the gastrocnemius muscle of NLRP3^−^ mice than WT mice. Moreover, melatonin therapy countered age-associated muscular changes and reduced the percentage of collagenous tissues deposition. Comparisons between groups are indicated in the graphs. * *p* < 0.05, ** *p* < 0.01 and *** *p* < 0.001.

**Figure 7:**
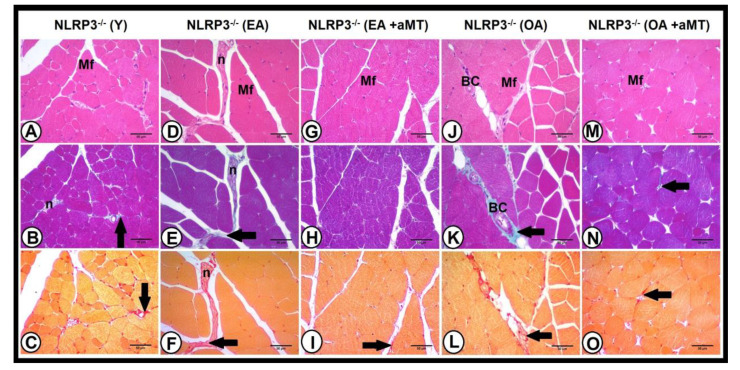
Impact of NLRP3 inflammasome and melatonin supplementation on the gastrocnemius muscle structure during aging. Photomicrographs of cross section of the gastrocnemius muscles of the young (Y), early aged (EA), early aged with melatonin (EA + aMT), old-aged (OA), and old-aged with melatonin (OA + aMT) NLRP3^−^ mice stained with H&E (**A**,**D,G**,**J**,**M**); Crossmon’s trichrome (**B**,**E**,**H**,**K**,**N**), and Van Gieson (**C**,**F**,**I**,**L**,**O**) stains. (**A**–**C**) Photomicrographs of the Y mice showing better skeletal muscle fibers (Mf) arrangement, with less percentage of collagenous fibers (arrows). (**D**–**F**) Photomicrographs of the EA animals revealing increase of the interstitial spaces, with significant induction of collagenous tissue accumulation (arrows) in interstitial spaces around nerve fibers (n). (**G**–**I**) Photomicrographs of the EA animals after melatonin therapy exhibiting the conservative effect of melatonin against age-associated muscular changes, where melatonin restored muscle fibers (Mf) architecture and reduced collagen (arrows) in interstitial spaces. (**J**–**L**) Photomicrographs of the OA mice illustrating increased collagen accumulation (arrows) in interstitial spaces around blood capillaries (BC). (**M**–**O**) Photomicrographs of OA mice after melatonin administration depicting the protective effect of melatonin on preserving muscle fibers (Mf) structure and inhibiting collagen deposition (arrows). Bar = 50 μm.

Light microscopy analysis of the gastrocnemius muscle revealed the normal muscular structure. The skeletal muscles were organized in bundles, which were separated by narrow interstitial spaces that support blood capillaries and nerve fibers, with less collagenous tissue percentage (2.87 ± 0.36%) in young WT mice (Figure 5A,C and Figure 6). With age, there was a widening of the interstitial spaces of the gastrocnemius muscle of EA (Figure 5D,F) and OA (Figure 5J,L) WT mice, with a significant increase of the collagen deposition (4.99 ± 0.82% and 6.97 ± 0.5%, respectively, *p* < 0.05) (Figure 6), associated with the presence of necrotic areas within the muscle fibers of OA animals. Melatonin administration, however, elucidated a protective effect against age-associated muscular alterations. It preserved muscular architecture, minimized interstitial spaces in both EA (Figure 5G,I) and OA (Figure 5M,O) WT animals, and significantly reduced the deposition of collagenous tissue fibers (1.62 ± 0.21% and 3.01 ± 0.42%, respectively, *p* < 0.05) (Figure 7).

Histological examination of the gastrocnemius muscle of young NLRP3^−^ mice showed better skeletal muscle fibers arrangement than those of young WT ones (Figure 7A,C), with less percentage of collagenous fibers (1.28 ± 0.24%) (Figure 6). Aging induced increase of the interstitial spaces in early aged (Figure 7D,F) and old-aged (Figure 7J,L) NLRP3^−^ mice, with significant induction of collagenous tissue accumulation (2.23 ± 0.25% and 4.21 ± 0.28%, respectively, *p* < 0.05). This increase in the percentage of the collagen with age was lower in the gastrocnemius muscle of NLRP3^−^ mice than WT mice (Figure 6). Furthermore, melatonin supplementation had a beneficial effect against age-related muscular damage, where it restored better muscular architecture, reducing the interstitial spaces in the gastrocnemius of the EA (Figure 7G,I) and OA (Figure 7M,O) NLRP3^−^ mice. Melatonin therapy significantly countered the percentage of collagenous tissues deposited in EA (1.01 ± 0.13%, *p* < 0.05) and OA (1.42 ± 0.18%, *p* < 0.05) mutant mice (Figure 6).

## 4. Discussion

Muscle aging is a complex process characterized by the accumulation of damage at molecular, cellular, and organ levels. Low-grade chronic and subclinical inflammation accompanies human aging has been termed “inflammaging” and it is considered a highly significant risk factor for age-related diseases [35]. Multiple studies support that inflammaging, induced by the innate immune system, plus oxidative damage induced by free radicals leads to a loss of muscular mass and strength, eventually resulting in frailty and sarcopenia [36,37,38]. It was shown that, at a morphological level, the gastrocnemius muscle of 12 months-old WT mice had a significant reduction in the overlapped actin/myosin length and of muscle fiber numbers, in addition to enhancement of connective tissue infiltration, both supporting contractile force loss with age [29,39]. In this study, light microscopical analysis revealed that gastrocnemius muscles of WT mice had widening of the interstitial spaces, with increase of collagen deposition as age advances. These changes were parallel with induced inflammatory and pro-apoptotic events. For the first time, the current study shows the upregulation of the three inflammatory-related miRs including miR-21, miR-146a, and miR-223 in aged skeletal muscle.

**Figure 8:**
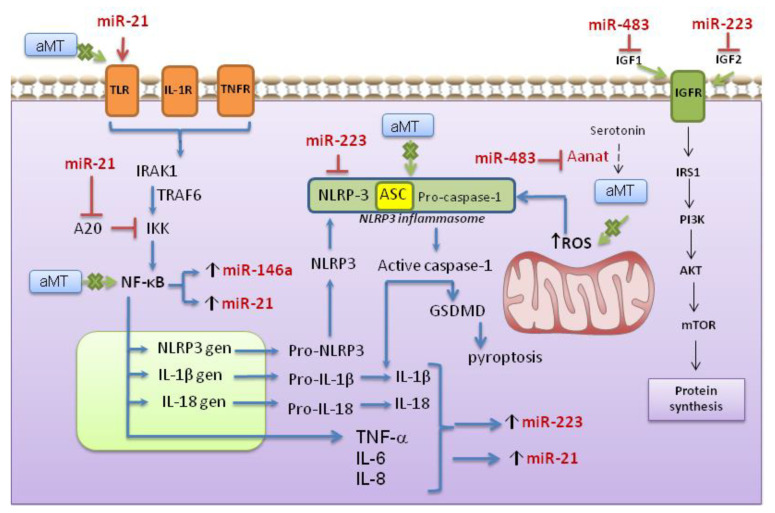
NF-κB pathway and NLRP3 inflammasome activation and role of some microRNAs. The priming signal acts through Toll-like receptor (TLR), specific receptors IL1-R, or TNFR that leads to the activation of NF-κB and subsequent production of pro-IL-1β, pro-IL-18, and pro-NLRP3. The second activation signal is provided by multiple events, including mitochondrial dysfunction, and reactive oxygen species (ROS) generation, among others, that damage mitochondria, opening the MPP releasing ROS and mtDNA to the cytosol. These signals activate NLRP3 inflammasome, that in turn causes caspase-1-depending IL-1β and IL-18 maturation. In conditions of high cytokine production, IL-1R and TNFR signaling activate the downstream kinase IRAK1 (interleukin-1 receptor-associated kinase 1), and TRAF6 (TNF receptor-associated factor 6), finally resulting in activation of NF-κB pathway that directly yields miR-146a and miR-21. miR-21 may act directly on TLRs and/or targeting A20 [15], activating NF-kB pathway. Moreover, the NF-κB-dependent TNF-α, IL-6, IL-8, as well as IL-1β and IL-18 production, the later ones activated by the NLRP3 inflammasome, increase miR-21 and miR-223 expression, the latter regulating NLRP3 inflammasome activation [6]. miR-223 and miR-483, acting through their target insulin-like growth factor (IGF)-2 and IGF-1, respectively, regulate the PI3K/AKT/mTOR pathway and blunt rates of protein synthesis [42,43]. Arylalkylamine-N-acetyltransferase (aanat) is the enzyme involved in melatonin synthesis and is a target for miR-483 [22]. The participation of melatonin is described in the text below.

miR-21 is one of the most studied miRs related to cellular senescence and inflammaging [18,40], and we found that it increased in EA and OA WT mice, whereas its expression did not change in NLRP3^−^ ones. As recently confirmed, miR-21 levels increased in senescent human umbilical vein endothelial cells (HUVECs) [41], in circulating extracellular vesicles of older people [41], as well as in plasma of older patients with frailty [17], whereas low miR-21 expression is related to health aging [18]. The pro-inflammatory effect of miR-21 may result from its ability to promote the NF-κB signaling pathway and subsequent NLRP3 inflammasome activation, acting directly on Toll-like receptors (TLRs) and/or through targeting A20 [15] (Figure 8). Increased levels of proinflammatory cytokines, such as TNF-α and IL-6, as well as ROS, induced miR-21 expression in primary myoblast cultures, contributing to defective muscle regeneration and resulting in muscle atrophy [41]. The positive correlation between miR-21 expression and pro-caspase-1 and pro-IL-1β here measured, supports miR-21 relationship with age-dependent inflammation and muscle damage in WT mice. Moreover, miR-21 may be an intrinsic positive regulator of NLRP3 inflammasome activity, as reported in septic mouse [15].

Thus, the reduction of pro-IL-1β due to the lack of NLRP3 may explain the drop of the expression of miR-21 in aged animals. Some authors suggest that, besides proinflammatory acting, miR-21 is also a profibrotic and apoptotic regulator [44,45,46], and may account for the positive correlation between miR-21 and Bax and p53 in WT mice. Interestingly, the positive correlation between miR-21 and Bax in WT mice was reversed in NLRP3^−^. These changes are accompanied by a higher expression of pro-apoptotic proteins Bax in early aged and old-aged mutant animals, and p53, and Bax/Bcl-2 ratio in early aged knock-out animals vs. WT of the same age, suggesting the increase of pro-apoptotic events in mutant mice. It is supposed that the balance between two processes, inflammation and apoptosis in skeletal muscle, is influenced by changes in miR-21 expression in NLRP3^−^ mice [47]. The increase of apoptotic markers with age may be due to activation of two apoptotic pathways; intrinsic apoptotic pathway, which is related to mitochondrial dysfunction and NLRP3 activation; and extrinsic apoptotic pathway that is mediated by TNFα binding to death receptor. The first pathway is related to the release of proapoptotic Bax, and the second one to the increased levels of caspase-3 and p53. The ablation of NLRP3 could trigger extrinsic apoptosis, increasing the levels of caspase-3 and p53. Some studies reported that inhibition of miR-21 promotes mitochondrial-mediated apoptosis [47,48]. Here we suppose that miR-21 acts through intrinsic apoptotic pathway, where a lower expression of miR-21 in ameliorated inflammatory conditions may reduce the antiapoptotic effect of miR-21, promoting apoptosis in the mutant mouse’s aged muscle. Apoptotic death of cells plays an important role in maintaining tissue integrity, and at this time it is not clearly documented whether prevention of apoptosis will lead to adaptive or maladaptive health outcomes for aged muscle. The increased miR-21 in young Y NLRP3^−^ mice is difficult to be explained at this time. miR-146a parallels changes in miR-21 increased the expression in early aged and old-aged WT mice, whereas it increased only in old-aged NLRP3^−^ animals. miR-146a takes great importance in muscular aging, because this miR participates in controlling mitochondrial activity during cellular aging, being considered both inflamma-miR and mito-miR [19,49], and supporting muscle senescence related to mitochondrial failure [50]. Several reports revealed that miR-146a increases its expression in response to high level of inflammation, related either to aging [17], Alzheimer’s disease [51], obesity [52], or other diseases, which are characterized by elevated oxidative stress and inflammation [14,20], and also was detected in cells of the high senescence-associated secretory (SASP) phenotype [53]. Although this miR has been related to inflammation and aging, few studies support its expression in tissues. Our results are in line with the evidence reported in gastrocnemius muscle from old (24 mo.) mice [54] and in human plasma [55]. Therefore, an increase in miR-146a production with age may be related to an increase in chronic inflammation through NF-κB pathway activation, which is supported here for the positive correlation found between miR-146a and pro-IL-1β in aged WT mice. Regarding apoptotic molecular markers, we found a positive correlation between the expression of miR-146a and pro-apoptotic proteins (Bax, caspase-3 and p53), which agrees with the pro-apoptotic effect of miR-146 elsewhere reported [49]. For example, these data are in line with the findings in mice with spinal muscular atrophy, where the loss of motor neurons was prevented by inhibition of miR-146a [56].

Similar to miR-21 and miR-146a, miR-223 increased in early aged and old-aged WT mice. Despite the fact that miR-223 targets NLRP3 and may have anti-inflammatory functions in myeloid cells, especially in macrophages and neutrophiles [21], its participation in different tissues is still unclear. Recent experiments found that exposure of adipocytes to TNF-α, which promotes the expression of classic proinflammatory molecules, increased intracellular miR-223 content [57]. The multi-omics study in a monocyte-macrophage cell line transfecting by pre-miR-223 altered osteoclastogenesis and macrophage differentiation via targeting several pathways, including the NF-κB one. Moreover, the changes in miR-223 expression were reported to influence the metabolic profile of cells, altering their apoptotic and proliferative conditions [58]. Aberrant expression of miR-223 was reported in cardiomyocytes subjected to hypoxia [59], as well as circulating miR-223 was increased in old frailty patients [17]. In contrast, the lack of NLRP3 prevents the expression of miR-223 in early aged and old-aged mutant mice. The positive correlations between miR-223 expression and pro-apoptotic Bax, p53, and caspase-3 reported here, are in line with that of Tang et al., who reported that inhibition of miR-223 improved cell viability and decreased the expression of Bax and caspase-3 in hypoxia-induced cardiomyocytes [59]. The reduced expression of miR-21 and miR-223 in NLRP3^−^ mice may depend, at least in part, on a reduced NF-κB activation due to the lack of a positive feedback by IL-1β. Additionally, the positive relation between miR-21, miR-146a and miR-223 expressions in WT mice and pro-apoptotic molecules found here, reflect age-associated increased in skeletal muscle apoptosis that we reported previously [29,60].

The drop of NLRP3 was associated with improving the skeletal muscle fibers arrangement and reduced collagen deposits compared with muscle of WT animals during aging. Some authors recently reported a role of NLRP3 inflammasome on age-related changes in skeletal muscle and heart [29]. We show that aging is associated with an increase of collagenous tissues deposition in the gastrocnemius muscle of mice, where fibrosis was less detectable in NLRP3^−^ mice than the WT ones [61,62]. Collagen infiltration reflects fibrosis [63], and its excessive content may depend on either of increased collagen synthesis and/or inadequate collagen degradation [64]. Collagen accumulation in the aged muscle displays two critical roles; (a) an eventual extracellular matrix increase, which minimizes the muscular lateral force transmission [65], and (b) an extracellular water increase in detriment of the intracellular water, which correlates with the reduced muscle performance during aging [66]. The reduction of collagen deposition in the aged muscles of NLRP3^−/−^ mice, compared with those of the WT mice, confirmed fibrosis attenuation after NLRP3 deletion [67]. These changes correspond to the decreased miR-21 and miR-223 expressions in NLRP3^−^ mice with age. Furthermore, our previous studies revealed that aging of WT and NLRP3^−^ mice was associated with a reduction of muscle fiber numbers, which was accompanied by compensated hypertrophy (increased cross-sectional area “CSA,” diameter and Feret’s diameter) of remaining fibers. These effects of age on muscle structure were significantly countered by melatonin supplementation [29,60].

Another exciting aspect to be emphasized here is the participation of miRs in the control of protein synthesis and/or loss of motor units of skeletal muscle [36]. Humans aged 70 and beyond show a reduction by 50% in the number of α-motor neurons together with a substantial reduction in muscle fiber size [68]. The imbalance in protein synthesis is potentially influenced by other miRs, including miR-21, miR-223, and miR-483. The TGF-β/myostatin/BMP pathway and the PI3K/AKT/mTOR pathway are involved in protein synthesis, and both are important for myogenic differentiation [36]. Taking into account that miR-21 also targets TGF-α and suppresses PI3K/AKT signaling [69], and miR-223 acts through their target insulin-like growth factor (IGF)-2 and regulates the PI3K/AKT/mTOR pathway [62]. The increase of both miRs with age can lead to inhibition of protein synthesis [36].

Concerning miR-483, different targets have been described, but very little is known about their host genes and their mechanisms in aging skeletal muscle tissue. Clokie et al. demonstrated that miR-483 extracted from the rat pineal gland acts as a suppressor of arylalkylamineN-acetyltransferase (aanat) mRNA expression, the key enzyme in melatonin synthesis [22]. It was reported that miR-483 is involved in the pathogenesis of Duchenne muscular dystrophy [70], alcohol-induced osteonecrosis of femoral head [71], and osteoporosis by targeting insulin-like growth factor-2 (IGF-2) through inhibiting osteoblast differentiation [72]. Moreover, high levels of miR-483 were detected in old adult´s plasma [17] and in vitro culture of human adipose mesenchymal stem cells correlated with adipogenic differentiation and cellular senescence mediated by targeting IGF1 [73]. Song et al. determined that miR-483 targets IGF1 and downregulated key proteins’ expression in the PI3K/AKT signaling pathway, suppressed both the proliferation and the differentiation of bovine muscle cells [43]. The increased expression of miR-483 in old-aged WT animals compared to the young ones was observed in this study, partly in line with Song’s results.

However, the function and expression of miR-483 may be context-dependent. The decreased expression of miR-483 and negative correlation between miR-483 and pro-caspase-1, caspase-3, and p53 levels in NLRP3^−^ mice may reflect the involvement of miR-483 in the regulation of age-related different signaling pathways, and it is barely beginning to be understood. Some studies have reported multiple and sometimes paradoxes effects of IGF1 on normal growth and senescence processes. For example, calorie restriction to inhibit IGF1 and mTOR has been demonstrated as an efficient anti-aging strategy by inducing autophagy [74]. Moreover, miR-483 was downregulated in pre-eclampsia and regulated endothelial progenitor cells by targeting IGF1 [42]. Further research is required to elucidate the effect of miR-483 on skeletal muscle with aging.

The mechanisms of the anti-oxidative and anti-inflammatory properties of melatonin include modulation of mitochondrial homeostasis, inhibition of free radicals’ production, and inhibition of the NF-kB/NLRP3 inflammasome activation [75,76]. Recently, it has been reported that melatonin supplementation improves cardiac mitochondrial ultrastructure altered by aging and have a cardio-protective role [26]. However, there are no reports about melatonin’s influence on miRs expression in aged skeletal muscle.

Here we report for the first time that oral administration of melatonin decreased the expression of miR-21, miR-146a, and miR-223 in WT mice with age. Furthermore, these reductions were accompanied by a lower expression of pro-caspase-1, pro-IL-1β, p53, and bax in WT mice with age. Melatonin can also inhibit TLRs, a common target for miR-21 too [77]. In addition to its anti-oxidative and anti-inflammatory effects, we observed here that melatonin has a capacity to reduce miR-21 expression in old skeletal muscle of mice. This finding may have a positive effect, and it is in line with the previously published evidence, where the inhibition of miR-21 attenuated fibrosis in the kidney, heart, and lungs [78,79]. Considering that melatonin decreases inflammation through the inhibition of the expression of NF-κB [75], it is not surprising the decreased miR-146a expression and reduced IL-1β production in treated early aged and old-aged WT mice, as well as old-aged NLRP3^−^ ones.

Regarding miR-223, melatonin reduced miR-223 expression in WT-aged mice, but in NLRP3^−^ animals, melatonin induced the expression of miR-223. The positive cellular environment due to the reduction of oxidative stress by melatonin administration can explain the reduction in miR-223 expression in WT mice with age. This result can take positive support that ablation of miR-223 effectively inhibited hypoxia-induced ROS generation and had a protective anti-apoptotic effect in cardiomyocyte H9c2 cells [59]. Mechanistically, the lack of NLRP3 abrogated the suppressive effect of melatonin on miR-223 expression during aging. Further studies for determining the mechanisms by which melatonin induced the upregulation of miR-223 expression in the lack of NLRP3, should be performed.

The increased expression of miR-483 with age may be related to the age-dependent decrease in melatonin production. Here we demonstrated that melatonin administration stimulated the expression of miR-483 in WT and NLRP3^−^ mice. These data may reflect the feedback-regulating mechanism on the melatonin synthesis in muscle.

Perhaps one of the limitations of this study was the reduced number of miRs analyzed, because other miRs may be involved in the mechanisms of protection and deterioration of muscle with age. Further studies in our group will point to analyze the relationship between additional miRs and pathways of muscle homeostasis to get more in-depth knowledge of the sarcopenia pathophysiology.

## 5. Conclusions

In this study and for the first time we reported that the miR-21, miR-146a, miR-223, and miR-483 expression change in the gastrocnemius muscle of mice during aging, which is modified in absence of NLRP3 inflammasome and melatonin treatment. The main outstanding data can be summarized as follows: (1) the increased expression of miR-21, miR-146a, and miR-223 in WT mice as age advances; (2) the positive relation between miR-21, miR-146a, and miR-223 expression and pro-inflammatory and pro-apoptotic molecules in WT mice, and (3) the suppression of NLRP3 inflammasome activation was related with decreased miR-21 and miR-223 levels, without changes in miR-146a. At morphological level we found that the lack of NLRP3 inflammasome improved the skeletal muscle fibers arrangement and reduced the collagen deposits compared with WT muscle during aging. For the first time, we showed that melatonin significantly reduced the expression of miR-21, miR-146a, and miR-223 in aged WT mice, increased miR-223 in NLRP3^−^ mice, and induced miR-483 expression in both mice strains. So, miRs and innate immunity converge, favoring age-dependent skeletal muscle impairment, whereas melatonin, which also decreased with age, may protect the muscle against aging.

## Data Availability

The data presented in this study are available on request from the corresponding author.

## Supplementary Materials

The following are available online at https://www.mdpi.com/article/10.3390/antiox10040524/s1. Table S1: The primers used for quantitative real-time PCR assay.
