# Supplementary material for: The Impact of Melatonin and NLRP3 Inflammasome on the Expression of microRNAs in Aged Muscle
Source: Antioxidants (Basel). 2021 Mar 27;10(4):524. doi: 10.3390/antiox10040524 (PMC8066875; doi:10.3390/antiox10040524)
Supplement: Supplementary file 1 [file antioxidants-10-00524-s001.pdf]

Supplementary Table 1. The primers used for quantitative real-time PCR assay.

Gene Symbol	Gene Description	Forward primer	Reverse primer
Caspase-1	Caspase-1	**CGTCTTGCCCTCATTATCTG**	**TCACCTCTTTCACCATCTCC**
il-1β	Interleukin-1beta	**TTCAGGCAGGCAGTATCAC**	**CAGCAGGTTATCATCATCATCC**
bcl2	B-cell lymphoma 2	**GCAGAGATGTCCAGTCAGC**	**GGCGATGTTGTCCACCAG**
bax	Bcl-2-associatedX protein	**GCCTCCTCTCCTACTTCGG**	**CCTCAGCCCATCTTCTTCC**
p53	Tumor protein p53	**GCTGCTCCGATGGTGATG**	**AGTGTGATGATGGTAAGGATAGG**
caspase 3	Caspase 3	**GCTGACTTCCTGTATGCTTAC**	**ATTCCGTTGCCACCTTCC**
β-actin	Beta-actin	**GCTGTCCCTGTATGCCTCTG**	**CGCTCGTTGCCAATAGTGATG**
